# The Amygdala as a Neurobiological Target for Ghrelin in Rats: Neuroanatomical, Electrophysiological and Behavioral Evidence

**DOI:** 10.1371/journal.pone.0046321

**Published:** 2012-10-10

**Authors:** Mayte Alvarez-Crespo, Karolina P. Skibicka, Imre Farkas, Csilla S. Molnár, Emil Egecioglu, Erik Hrabovszky, Zsolt Liposits, Suzanne L. Dickson

**Affiliations:** 1 Department of Physiology, Institute of Neuroscience and Physiology, The Sahlgrenska Academy at the University of Gothenburg, Gothenburg, Sweden; 2 Laboratory of Endocrine Neurobiology, Institute of Experimental Medicine, Hungarian Academy of Sciences, Budapest, Hungary; 3 Department of Pharmacology, Institute of Neuroscience and Physiology, The Sahlgrenska Academy at the University of Gothenburg, Gothenburg, Sweden; 4 Department of Neuroscience, Faculty of Information Technology, Pázmány Péter Catholic University, Budapest, Hungary; Hosptial Infantil Universitario Niño Jesús, CIBEROBN, Spain

## Abstract

Here, we sought to demonstrate that the orexigenic circulating hormone, ghrelin, is able to exert neurobiological effects (including those linked to feeding control) at the level of the amygdala, involving neuroanatomical, electrophysiological and behavioural studies. We found that ghrelin receptors (GHS-R) are densely expressed in several subnuclei of the amygdala, notably in ventrolateral (LaVL) and ventromedial (LaVM) parts of the lateral amygdaloid nucleus. Using whole-cell patch clamp electrophysiology to record from cells in the lateral amygdaloid nucleus, we found that ghrelin reduced the frequency of mEPSCs recorded from large pyramidal-like neurons, an effect that could be blocked by co-application of a ghrelin receptor antagonist. In *ad libitum* fed rats, intra-amygdala administration of ghrelin produced a large orexigenic response that lasted throughout the 4 hr of testing. Conversely, in hungry, fasted rats ghrelin receptor blockade in the amygdala significantly reduced food intake. Finally, we investigated a possible interaction between ghrelin's effects on feeding control and emotional reactivity exerted at the level of the amygdala. In rats allowed to feed during a 1-hour period between ghrelin injection and anxiety testing (elevated plus maze and open field), intra-amygdala ghrelin had no effect on anxiety-like behavior. By contrast, if the rats were not given access to food during this 1-hour period, a decrease in anxiety-like behavior was observed in both tests. Collectively, these data indicate that the amygdala is a valid target brain area for ghrelin where its neurobiological effects are important for food intake and for the suppression of emotional (anxiety-like) behaviors if food is not available.

## Introduction

The stomach-derived hormone, ghrelin [1], has emerged as a prominent gut-brain signal, whose integrated CNS effects are important for feeding control. Ghrelin has neurobiological effects that extend beyond food intake [2] to include behaviors linked to reward, learning, memory, cognition and emotional reactivity/mood [3]–[11]. Central ghrelin signaling involves a dedicated receptor, the growth hormone secretagogue receptor (GHS-R, subtype 1A) [12], whose CNS distribution includes relevant brain areas such as the hypothalamus, brainstem and some limbic areas [13], [14]. In rats, feeding behavior can be driven by microinjection of low doses of ghrelin into many of these sites, including discrete hypothalamic (e.g. arcuate nucleus, lateral hypothalamus and paraventricular nucleus) [15]–[17] and brainstem (by 4^th^ ventricular administration [18]) areas, as well as into key mesolimbic areas linked to reward, such as the ventral tegmental area and nucleus accumbens [5]–[7] There are indications that ghrelin can also drive feeding behavior when administered into brain areas involved in emotional reactivity/mood, such as the hippocampus and dorsal raphe nucleus [19].

Another key brain area linking feeding control with emotional reactivity/mood is the amygdala. The amygdala is involved in memory and emotion processing but it also plays an important role in the modulation of reward, learning, and attention [20]. As reviewed elsewhere [21], the amygdala is closely interconnected with hypothalamic, midbrain/striatum, limbic and cortical pathways involved in feeding control. In man, activity in these brain areas (including the amygdala) in response to visual food cues, is increased both by fasting [22] (i.e. when circulating ghrelin levels are highest) and by peripheral ghrelin administration [23]. Surprisingly, however, the amygdala remains rather unexplored as a target for ghrelin's orexigenic effects. This may reflect the fact that studies describing the distribution of GHS-R in rodent brain, do not document expression in the amygdala [13], [14]. However, its presence is implied from studies showing that intra-amygdala injection of ghrelin to rats influences behaviors linked to memory, learning and emotional reactivity/mood [9], [19], [24]–[26]. An overarching hypothesis explored here is that ghrelin's neurobiological role extends to direct actions at the level of the amygdala that integrate ghrelin's well-documented orexigenic role with effects on emotional reactivity/mood.

Studies to date linking ghrelin with emotional reactivity/mood (especially anxiety-like behavior) are not altogether in agreement. For example, acute ghrelin injection has been shown to both increase [27], [28] and decrease [10] anxiety-like behavior in the elevated plus maze (EPM) test. The response may also be dependent upon the chronicity of treatment, the dose and the route/target site of injection [11], [19], [29]. One interesting possibility, to be explored here, is that ghrelin's effects in the EPM test are linked to its role in appetitive behavior. In other words, immediately following a single ghrelin injection, rats normally express feeding-related behaviors; subsequent performance in the EPM test could therefore be influenced by food availability.

In the present study, we explored the amygdala as a target for ghrelin's behavioral effects linked to appetite control and emotional reactivity/mood. Given the uncharted nature of the amygdala GHS-R distribution, we first sought to describe the presence and neuroanatomical distribution of this receptor in the amygdala of the rat. Next, we explored cellular electrophysiological responses of amygdala cells to ghrelin and ghrelin antagonists. Finally, we determined the effects of intra-amygdala ghrelin injection on food intake and also on anxiety-like behavior in both the EPM and open field tests, that takes into account food availability immediately prior to the tests.

## Materials and Experimental Methods

### Ethics Statement

All these studies were carried out with ethical permissions from the Animal Welfare Committee (IEM, Budapest) and from the Animal Ethics committee at the University of Gothenburg and in accordance with legal requirements of the European Community.

### Animals

For electrophysiology and *in situ* hybridization studies, male Wistar rats were used. They were obtained from a local colony bred at the Medical Gene Technology Unit of the Institute of Experimental Medicine (IEM, Budapest, Hungary). Acute amygdala slices from juvenile (2–4 weeks old) rats were used for electrophysiology, whereas the *in situ* hybridization detection of GHS-R mRNA was carried out on tissues sections of adult (8 week old) rats.

For studies investigating food intake and anxiety-like behaviour, adult male Sprague-Dawley rats (200–250 g, Charles River, Sulzfeld, Germany) were used. All animals were housed in a 12 hr light/dark cycle and maintained under controlled temperature (20–22°C) with free access to regular chow and water, except where indicated otherwise.

### Drugs

Ghrelin (Tocris, Bristol, UK) was dissolved in physiological saline. For food intake and anxiety studies, the dose of ghrelin selected for intra-amygdala injection was 0.3 and 1 µg/rat, microinjected in a volume of 0.2 µl saline. The 1 µg dose was used previously to drive food intake when administered to the ventral tegmental area or nucleus accumbens, while the lower dose was sub-threshold [6], [30]. JMV2959 (AEZS-123, gift from AeternaZentaris GmBH, Frankfurt, Germany), a GHS-R1A antagonist [31], was dissolved in physiological saline and microinjected in the amygdala at the doses 2 or 10 µg/rat in a volume of 0.2 µl. All injections of ghrelin/saline took place between 9.00 and 10.00 am. For electrophysiological studies, the selected dose was 4 µM for ghrelin [11], [32] and 10 µM for JMV2959 [33].

### Localization of GHS-R mRNA in the Amygdala by *in situ* Hybridization

Here we sought to provide a clear account of the distribution of GHS-R in the amygdala of the rat. Pre-hybridization, hybridization and post-hybridization procedures were performed on slide-mounted 12 µm frozen sections [34]. The generation and use of the ^35^S-labeled cRNA probe to GHS-R mRNA (targeting bases 237–1073 of the mouse sequence; NM_177330) was described previously [35]. To enhance sensitivity, high probe, dextran sulphate and dithiothreitol concentrations were added to the hybridization solution [36] Sense RNA transcripts were labelled and used similarly on control sections that were processed in parallel. Following post-hybridization, the slides were coated with Kodak NTB nuclear track emulsion (Kodak, Rochester, NY, USA; diluted 1∶1 with MQ water) and exposed for 4 weeks. The autoradiographic images were developed with Kodak processing chemicals, counterstained with toluidine blue, dehydrated and cover-slipped. Digital photomicrographs of bright-field autoradiographic images were prepared with an AxioCam MRc 5 digital camera mounted on a Zeiss AxioImager M1 microscope, using the AxioVision 4.6 software (Carl Zeiss, Göttingen, Germany). The images were processed with the Adobe Photoshop 7.1 software at 300 dpi resolution.

### Determination of the Effects of Ghrelin or a Ghrelin Receptor Antagonist on the Electrophysiological Response of Single Amygdala Cells Recorded *In Vitro*

#### Brain Slice Preparation

Rats were anesthetized using isoflurane inhalation. The brain was removed rapidly and immersed in ice-cold cutting solution (in mM: sacharose 252.0, KCl 2.5, NaHCO_3_ 26.0, CaCl_2_ 2.0, MgCl_2_ 2.0, NaH_2_PO_4_ 1.25, and glucose 10) bubbled with a mixture of 95% O_2_ and 5% CO_2_. 400-µm-thick coronal slices were then prepared containing the lateral amygdala with a VT-1000S vibratome (Leica GmBH, Wetzlar, Germany) in ice-cold oxygenated cutting solution. The slices were bisected along the midline and transferred into artificial cerebrospinal fluid (aCSF, in mM: NaCl 126.0, KCl 2.5, NaHCO_3_ 26.0, CaCl_2_ 2.0, MgCl_2_ 2.0, NaH_2_PO_4_ 1.25, and glucose 10) saturated with O_2_/CO_2_, and kept in it for 1 hr to equilibrate. Equilibration started at 33°C and was allowed to cool to room temperature. Electrophysiological recordings were carried out at 33°C, during which the brain slices were oxygenated by bubbling the aCSF with O_2_/CO_2_ gas. Axopatch 200B patch clamp amplifier, Digidata-1322A data acquisition system, and pCLAMP 9.2 software (Molecular Devices Co., Sunnyvale, CA) were used for recording. Cells were visualized with a BX51WI IR-DIC microscope (Olympus Co., Tokyo, Japan). The patch electrodes (OD = 1.5 mm, thin wall; Garner Co., Claremont, CA) were pulled with a Flaming-Brown P-97 puller (Sutter Instrument Co., Novato, CA) and polished with an MF-830 microforge (Narishige, Tokyo, Japan). After control recording, the slices were treated with various drugs for 10 min and the recording repeated for 250 sec. Each neuron served as its own control when effects of the drugs were evaluated.

#### Whole-Cell Patch-Clamp Experiments

The cells were voltage clamped at a −70 mV holding potential. The pipette solution contained (in mM): HEPES 10.0, K-gluconate 136.0, KCl 4, EGTA 5.0, CaCl_2_ 0.1, Mg-ATP 4.0, and Na-GTP 0.4 (pH 7.3 with NaOH). The resistance of the patch electrodes was 3–5 MΩ. Spike-mediated transmitter release was blocked in all experiments by adding the voltage-sensitive Na-channel inhibitor tetrodotoxin (TTX, 750 nM; Tocris) to the aCSF 10 min before control miniature excitatory postsynaptic currents (mEPSCs) were recorded. Picrotoxin (100 µM; Sigma, St. Louis, MO) was also used in all experiments in the aCSF to block GABA_A_-receptor mediated inhibitory postsynaptic currents. After establishing whole-cell clamp configuration the mEPSCs were recorded. After this, rat ghrelin (4 µM) or the ghrelin receptor antagonist JMV2959 (10 µM) were applied for 10 min in the aCSF and the mEPSCs were recorded.

### Effects of Intra-Amygdala Injection of Ghrelin on Feeding Behavior and Anxiety-Like Behavior

#### Surgery for Intra-Amygdala Injection

**Figure 1:**
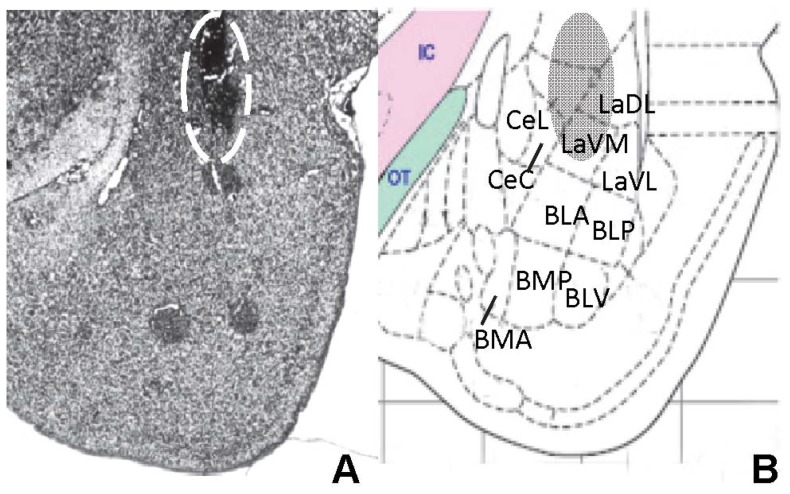
Histological verification of the location of the injection cannula in the lateral amygdaloid nucleus. A: Photomicrograph of a 40 µm counterstained coronal section of rat brain at level Bregma −3.3, illustrating the injection site. B: Schematic representation of the amygdala according to the rat brain atlas [37]. The shadow area outlines the region defined as the lateral amygdaloid nucleus. Scale bar = 1 mm. Abbreviations: BLA (basolateral amygdaloid nucleus, anterior), BLP (basolateral amygdaloid nucleus, posterior), BMP (basomedial amygdaloid nucleus, posterior), BMA (basomedial amygdaloid nucleus, anterior), CeC (central amygdaloid nucleus, central), CeL (central amygdaloid nucleus, lateral), LaDL (lateral amygdaloid nucleus, lateral), LaVL (lateral amygdaloid nucleus, ventrolateral), LaVM (lateral amygdaloid nucleus, ventromedial), OT (optic tract).

The rats were acclimatized to the animal facility for at least 7 days before commencing surgery. They were anesthetized with isoflurane and placed in a stereotaxic apparatus for catheter placement. A stainless steel guide cannula (26 gauge; Plastics One, Roanoke, VA, USA) was positioned in the amygdala using the following stereotaxic coordinates from bregma: anteroposterior – 2.8 mm, lateral 4.8 mm, dorsoventral – 6.6 mm), according to a rat brain atlas [37]. These coordinates correspond to an area with dense expression of GHS-R, in the *in situ* hybridization studies. The guide cannula was fixed to the skull with dental acrylic cement and closed with an obturator. The rats were placed in single cages and allowed to recover for one week before the experiment. To facilitate focal injection of a low volume (0.2 µl) we used a Hamilton syringe connected to a 30-gauge needle that extended 2 mm beyond the tip of the guide cannula. An infusion pump allowed the slow delivery of the drug (over 1 min) to conscious rats. The injectors remained in place for one additional minute to avoid reflux of the solution before being removed and replaced with the obturator. The correct placement of the cannula was verified *post mortem* by injecting 0.2 µl of India blue ink and assessing the selective dye distribution on brain sections (Fig. 1).

#### Effects of Intra-Amygdala Injection of Ghrelin or a Ghrelin Receptor Antagonist on Food Intake

These experiments were designed to determine whether ghrelin action at the level of the amygdala is important for food intake in rats. Ghrelin (0.3 or 1 µg/rat) or vehicle were injected unilaterally into the amygdala of satiated rats and food intake measured at 1, 2, 4 and 24 hr after injection. A counterbalanced design was used where each rat received all the possible treatments, saline, ghrelin 0.3 µg and ghrelin 1 µg, each separated by a minimum of 48 hr. Using an identical experimental design, food intake was also measured after intra-amygdala injection of JMV2959 (2 or 10 µg/rat) or vehicle to rats that had been fasted overnight.

#### Effects of Intra-Amygdala Injection of Ghrelin on Anxiety-Like Behavior in the EPM and Open Field Tests

**Figure 2:**
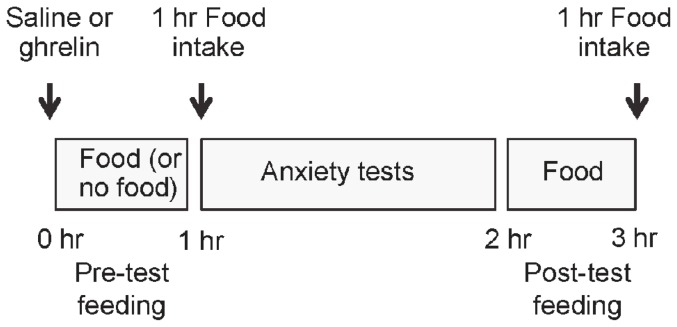
Protocol for anxiety-related studies. Fed rats were injected with saline or ghrelin directly into the amygdala at time zero. In the FOOD ACCESS paradigm, rats were allowed access to food during the first hour after injection whereas food access was denied in the FOOD WITHHELD paradigm. After this, all rats underwent tests exploring anxiety-like behaviour, first in the EPM test (5 min) and then in the open field test (40 min). Afterwards all the rats were returned to their home cages and post-test food intake measured for 1 hr (corresponding to time 2–3 hr after injection).

The experimental protocol is illustrated schematically in Fig. 2. Satiated rats were microinjected with either ghrelin (1 µg/rat) or saline directly into the amygdala. Two paradigms were explored. In the FOOD WITHHELD paradigm, rats were denied access to chow during the 1 hr period immediately after the intra-amygdala injection, whereas food was freely available (and measured) during this period in the FOOD ACCESS paradigm. After this hour, anxiety-like behavior was explored, first in the EPM test and then, 10 min later, in the open field test. Finally, the rats were returned to their home cages with free access to food during which time their intake was measured. This food measurement serves to verify biological effects (i.e. food intake) of intra-amygdala ghrelin. It was also used to indicate that 1 hr of denied food access (commencing around 9.00 to 10.00 am for all rats) does not cause sufficient metabolic deficit to induce a strong feeding response.

The EPM apparatus consisted of two open arms (50×10 cm^2^) made of black PVC (polyvinyl chloride), crossed by two closed arms of the same size but with walls of 40 cm high. All the arms emerged from a central platform placed 50 cm above the floor. Under dim light (around 100 lux over the open arms and 60 lux over the closed arms) every rat was placed in the central platform and they were allowed to freely move in the whole apparatus during 5 min. The EPM apparatus was cleaned between each trial. The rat behavior was recorded by a video camera during this time for subsequent determination of the following parameters: the number of entries in the open arms, the number of entries into the closed arms (an entry was counted when the four paws were placed on the respective arm) and the time spent in the open arms and in the closed arms.

At the end of EPM testing, the rats were undisturbed for 10 min before being placed in sound attenuated locomotor activity boxes (40 min) for the open field test. These boxes, measuring 1×1×0.5 m contained an inner Plexiglas chamber (0.7×0.7×0.35 m) and they were equipped with an automatic system integrated by two rows of eight photocells on each side, that allow to detect movements (Kungsbacka Mät- och reglerteknik, Sweden). Under dim light (40 lux) the locomotor activity is assessed by registration of the breaking of a sequence of beams. The peripheral activity is measured by the photocells closed to the corners while the central activity is registered by the central 6 photocells on each side. The photocells in the upper row measure rearing. Finally they were returned to their home cage for further food intake measurement (taken during the 2–3^rd^ hr after ghrelin/saline injection).

### Statistical Analysis

In the electrophysiology studies each experimental group contained 8–10 recorded cells from six to seven animals. Recordings (250 sec) were stored and analysed off-line. Event detection was performed using the Clampfit module of the PClamp 9.2 software (Molecular Devices Co.). Group data were expressed as mean ± SEM and percentage change in the frequency of the mEPSCs due to the application of the ghrelin or the JMV2959 was calculated. Statistical significance was analysed using Student's *t* test or ANOVA followed by Newman-Keuls (NK) test (GraphPad Software, Inc., GraphPad, San Diego, CA) and considered at *P*<0.05.

For the behavioral experiments the data were analyzed in IBM SPSS Statistics 9. The “P" values <0.05 were considered statistically significant. All the data are presented as mean ± Standard Error of the Mean (SEM).

## Results

### *In situ* Hybridization Detection of GHS-R mRNA in the Amygdala

**Figure 3:**
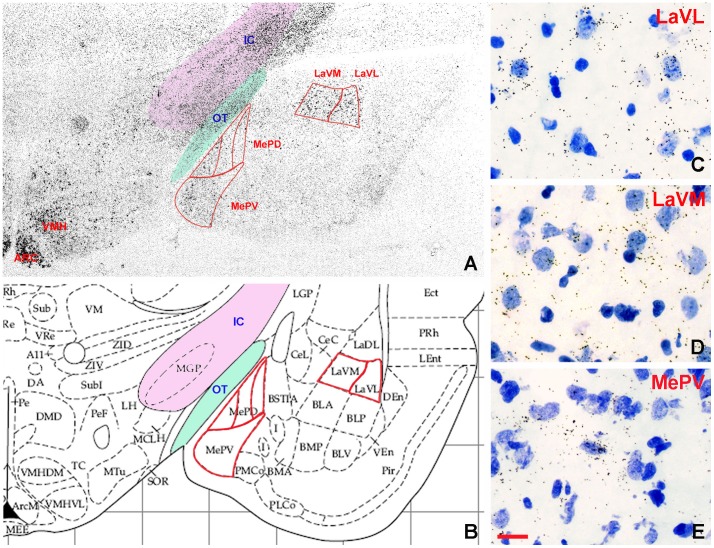
*In situ* hybridization detection of GHSR mRNA in the rat amygdala. The autoradiographic detection of the isotopic *in situ* hybridization probe to GHSR mRNA revealed GHSR mRNA expression in distinct neurons of the amygdala. Note in A that the highest levels of expression could be observed in the ventrolateral (LaVL) and ventromedial (LaVM) parts of the lateral amygdaloid nucleus and in the posteroventral part of the medial amygdaloid nucleus (MePV). The parcellation and nuclear structure of the amygdala is shown in B. Also note the heavy GHSR mRNA signal in the arcuate (ARC) and ventromedial (VMH) nuclei. High-power photomicrographs (C–E) were amplified from various regions of the amygdala shown in A (cresyl violet counterstaining). Scale bars = 500 µm in A and B and 13 µm in C–E.

The autoradiographic detection of *in situ* hybridization signal revealed the presence of GHS-R mRNA expressing neurons in distinct neurons of the amygdala. The signal strength was variable in different amygdaloid nuclei, with the highest levels of expression in the ventrolateral (LaVL) and ventromedial (LaVM) parts of the lateral amygdaloid nucleus and somewhat lower levels in the medial amygdaloid nucleus, including its posteroventral part (MePV) (Fig. 3). Silver grain clusters were entirely absent from sections hybridized with the sense RNA transcripts (not shown).

### Whole-Cell Patch-Clamp Recordings from Cells in the Lateral Amygdala: Effects of Ghrelin and a Ghrelin Receptor Antagonist

**Figure 4:**
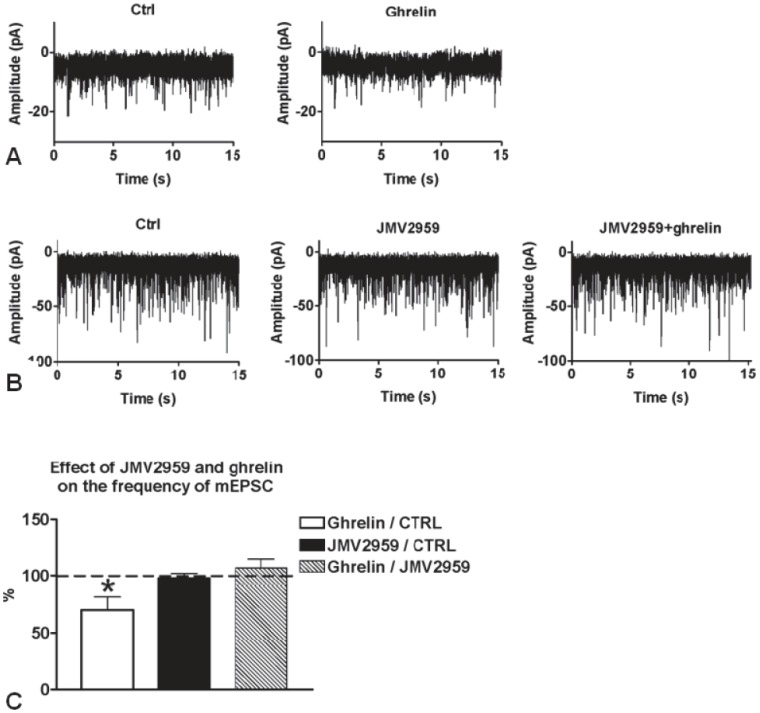
Whole-cell patch clamp recordings of mEPSCs in large pyramidal-like neurons of the lateral amygdala. A) Application of ghrelin (4 µM) in the extracellular solution decreased the frequency of the mEPSCs. B) Extracellular administration of the ghrelin receptor antagonist JMV2959 (10 µM), blocked this effect of ghrelin. C) Histogram shows the relative percentages of mEPSC frequencies. *P<0.05.

Neurons of the lateral amygdala could be categorized into two basic cell types: large, pyramidal-like neurons and smaller, non-pyramidal-like neurons [38]. To test the hypothesis that ghrelin directly influences excitatory neurotransmission in the lateral amygdala, ghrelin effects on mEPSCs were recorded from the two different cell types in the presence of TTX and picrotoxin. Small neurons showed no response; the frequency of the mEPSCs presented no significant change upon ghrelin (4 µM) administration (not shown). In contrast, when ghrelin was applied to the large, pyramidal-like neurons, it decreased the frequency of the mEPSCs significantly (70.3±11.4% of the control; Fig. 4A and 4C). In addition, when ghrelin was administered in the presence of the GHS-R1A antagonist, JMV2959 (10 µM), mEPSCs frequency remained unaltered (106.8±7.82% of the mEPSC frequency obtained with JMV2959 alone; Fig. 4B and 4C). Application of JMV2959 alone did not affect the frequency of the mEPSCs of the large neurons (98.0±4.03% of the control; Fig. 4B and 4C).

### Intra-Amygdala Administration of Ghrelin: Effects on Chow Intake

**Figure 5:**
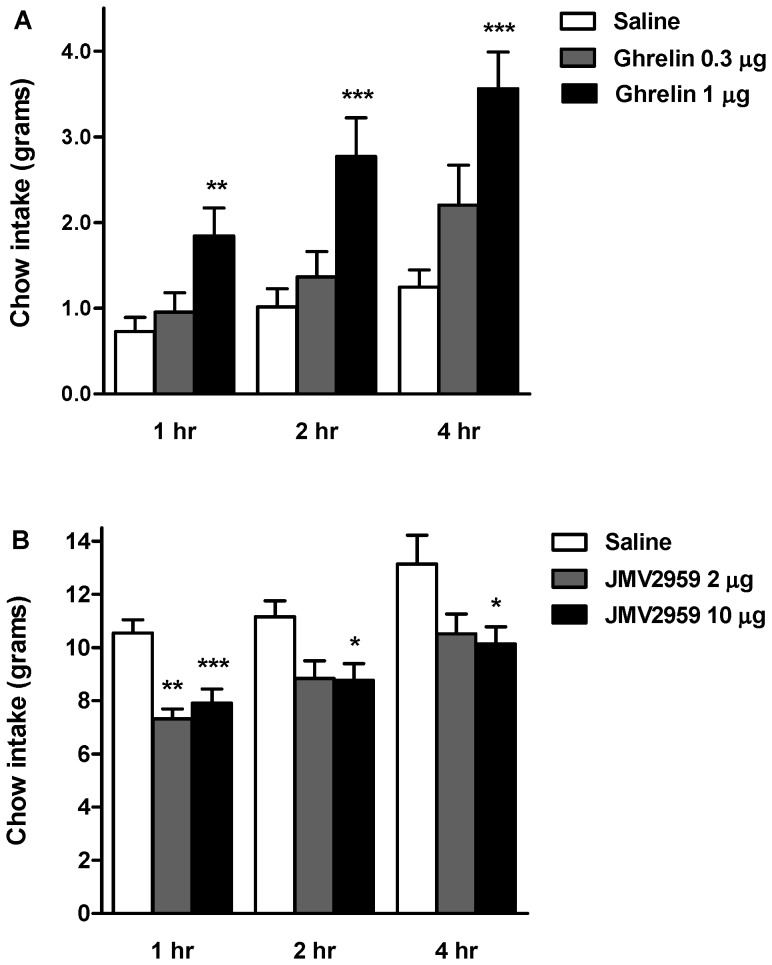
Effects of intra-amygdala administration of ghrelin or a ghrelin antagonist (JMV2959) on cumulative food intake. Normal chow intake was measured at 1, 2, 4, and 24 hr following unilateral intra-amygdala injection of either (A) ghrelin (0.3 and 1 µg) to freely-fed rats or (B) JMV2959 (2 or 10 µg) to overnight fasted rats. Data are expressed as mean ± S.E.M. *P<0.05 **P<0.01 ***P<0.001, vs. saline. Paired sample t-test, SPSS.

In satiated rats, unilateral administration of the higher ghrelin dose (1 µg) to the amygdala induced a >2.5 fold increase in food intake relative to saline-treated controls, measured at 1, 2, and 4 hr after the microinjection. The lower dose (0.3 µg) was without effect (Fig. 5A). By contrast, intra-amygdala injection of JMV2959 to overnight fasted rats significantly decreased chow intake, measured at 1, 2, 4 and 24 hr post-injection. A JMV2959 dose of 10 µg was effective at all time points, whereas the lower dose of 2 µg only suppressed food intake at the 1 hr time point (Fig. 5B).

### Intra-Amygdala Administration of Ghrelin: Effects on the Anxiety-Like Behavior

**Figure 6:**
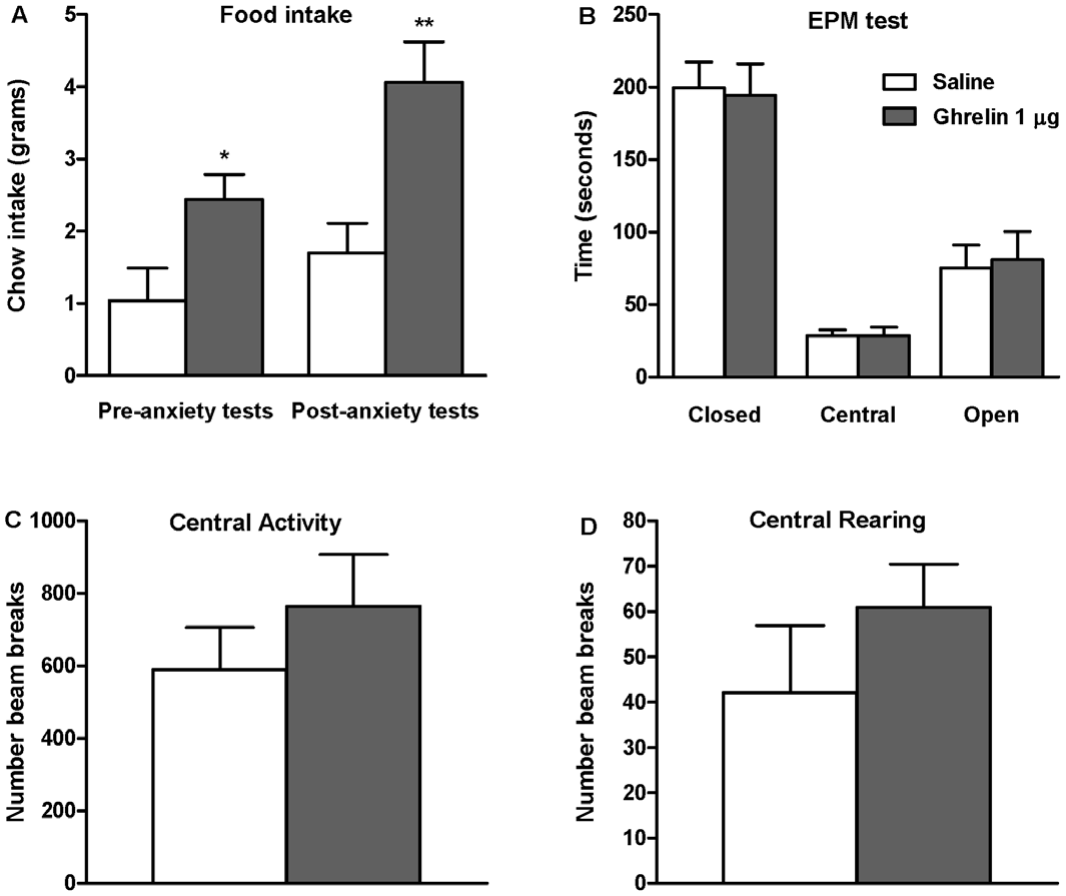
Effects of intra-amygdala administration of ghrelin on anxiety-like behavior in rats given access to food. In rats given access to food during the first hour after intra-amygdala injection (FOOD ACCESS), ghrelin increased food intake relative to saline controls (g of chow), both during this hour and during the 1 hr measurement taken after the anxiety tests (A). In this paradigm there was no effect of ghrelin (relative to saline controls) on anxiety-like behavior in either the EPM test (time spent in the open arm; B) or the open field test (central activity or central rearing; C, D respectively). *P<0.05 **P<0.01, vs. saline. Independent samples t-test, SPSS.

**Figure 7:**
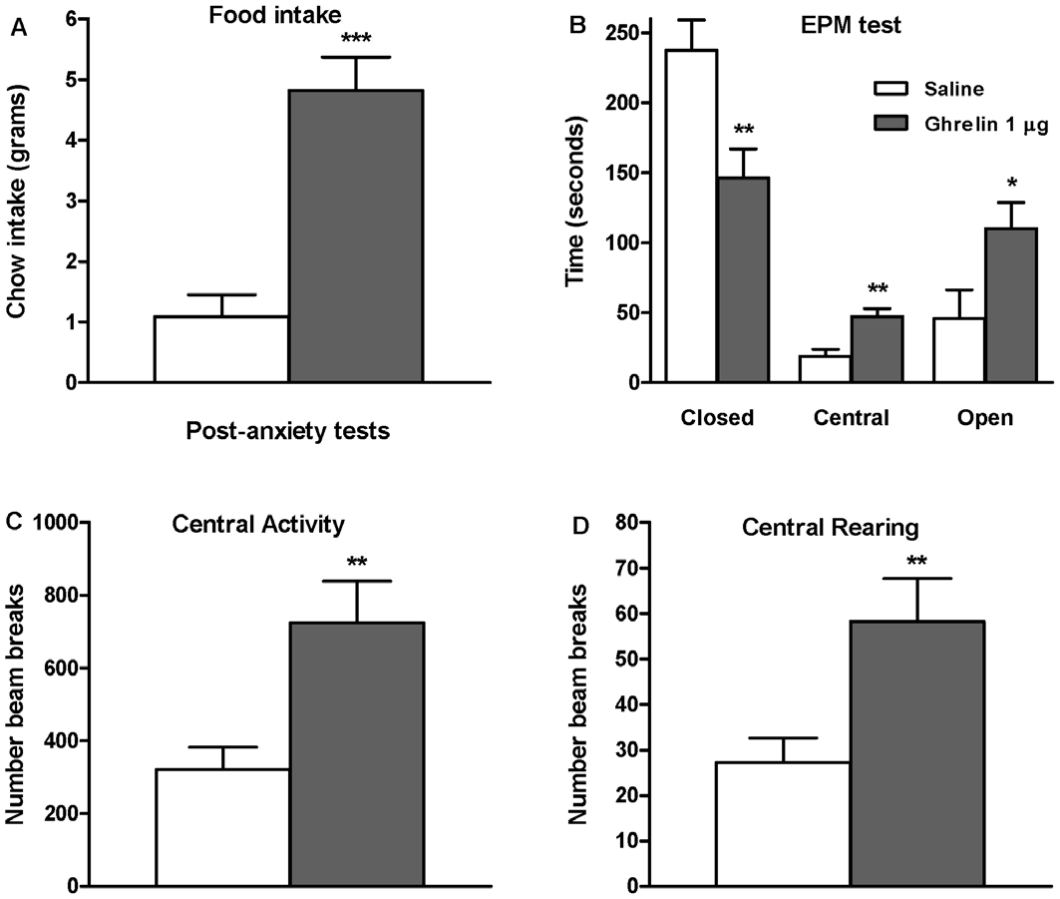
Effects of intra-amygdala administration of ghrelin on anxiety-like behavior in rats denied access to food. In this “FOOD WITHHELD" paradigm rats were denied access to food during the first hour after intra-amygdala injection. (A) An orexigenic response to intra-amygdala ghrelin injection was detected when animals were returned to their home cages after the anxiety testing. Intra-amygdala ghrelin injection decreased anxiety-like behavior relative to saline controls, reflected by an increase in the amount of time spent in the open arms in the EPM test (B) and by the increase in central activity (C) and central rearings (D) in the open field test. *P<0.05 **P<0.01, ***P<0.001, vs. saline. Independent samples t-test, SPSS.

First, we used the EPM test to explore the effects of intra-amygdala injection of ghrelin on emotional reactivity (anxiety-like behavior) in rats allowed access to food during the first hour following injection (FOOD ACCESS paradigm; Fig. 6), and also in rats that did not have access during this hour (FOOD WITHHELD paradigm; Fig. 7). Intra-amygdala ghrelin injection induced a robust feeding response, measured both during the hour period prior to the anxiety test (measurable only in the FOOD ACCESS paradigm; Fig. 6A) and during the hour after the anxiety test, during which all rats were allowed food intake *ad libitum* (Fig. 6A, 7A). In the FOOD ACCESS paradigm we did not detect any change in anxiety-like behavior between ghrelin-treated (1 µg) and saline-treated groups, as there was no significant difference in the time spent in the open arms (Fig. 6B) or the number of entries into the open arm (saline: 4.8±1.2 vs. ghrelin: 5.7±1.4). However, in the FOOD WITHHELD paradigm, intra-amygdala ghrelin decreased anxiety-like behavior, reflected by an increase in both the time spent in the open arm (Fig. 7B) and the number of open arm entries (saline: 2.4±0.9vs. ghrelin 1 µg: 6.4±0.7; P<0.006).

The effects of intra-amygdala ghrelin injection on anxiety-like behavior were further explored/validated in the open field test: ghrelin increased both central activity and central rearing in the FOOD WITHHELD paradigm (Fig. 7C, 7D). By contrast, no such effects of intra-amygdala ghrelin were observed in the FOOD ACCESS paradigm (Fig. 6C, 6D). Total locomotor activity (i.e. beam breaks) measured during the open field test was similar after intra-amygdala ghrelin administration in both experiments (FOOD ACCESS: saline 399± 44 vs. ghrelin 506±79 and FOOD WITHHELD: saline 383±49 vs. ghrelin 435±37; P = 0.26 and P = 0.42 respectively), indicating that the increased activity in the central area in the FOOD ACCESS paradigm is likely linked to emotional reactivity rather than a general locomotor response.

## Discussion

In the present study we explored the effects of ghrelin on the electrophysiological activity of amygdaloid pyramidal neurons and extended these findings to behavioral outputs of amygdala: food intake and anxiety-like behavior. First we validated the amygdala as a target for ghrelin by demonstrating the presence of ghrelin receptor (GHS-R) mRNA selectively in the lateral and medial amygdaloid nuclei of the rat. Using this information we performed whole cell patch clamp studies from amygdala cells located in the lateral amygdala, where GHS-R is most abundant. We found that ghrelin suppressed the electrical activity (i.e. a decreased number of mEPSCs) of pyramidal-like neurons in this region. Again, guided by localization of GHS-R, we demonstrated that intra-amygdala ghrelin injection (via catheters directed towards areas where GHS-R is most abundant) induced a robust feeding response. These data suggest that ghrelin's neurobiological effects in this brain area are linked to food intake, as shown previously for almost all other GHS-R-expressing brain areas studied, as reviewed previously [39]–[41]. Interestingly, in the EPM test, acute ghrelin injection into the amygdala increased the amount of time spent in the open arms (consistent with a decrease in anxiety-like behavior), but only in rats that were prohibited from feeding during the initial hour after injection. Thus, ghrelin-responsive networks are present in the amygdala that contribute to ghrelin's orexigenic effects and that appear to suppress anxiety-like behavior when food is not immediately available.

To our knowledge, this is the first study to describe the presence of GHS-R mRNA in the amygdala of the rat, by *in situ* hybridization, and describe its distribution. Specifically, we found that expression was most abundant in the lateral amygdaloid nucleus (Fig. 3) with lower expression levels in the medial nucleus. Accordingly, the electrophysiological studies that target this lateral nucleus demonstrate that ghrelin decreases the frequency of mEPSCs recorded, indicating that ghrelin reduces synaptic transmission in this area. Specificity of this effect to GHS-R1A was verified by the fact that co-application of a GHS-R1A antagonist with ghrelin abolished ghrelin's electrophysiological effects. Given that the lateral amygdaloid nucleus is considered to be functionally linked to affective motivated behavior, connected to the nucleus accumbens [42], it is well placed to receive and contribute to ghrelin's effects on food intake and food-motivated behavior. Indeed, this subnucleus of the amygdala, that we now demonstrate is ghrelin-responsive, is known to be important for appetitive learning and for assigning emotional and motivational significance to environmental cues [43]–[45].

Previous studies have shown that hyper-excitability of pyramidal-like neurons in the lateral amygdaloid nucleus is linked to anxiety-like behavior [46]. Conversely, it has also been shown that a reduction of this activity, for example via activation of inhibitory neuropeptide Y-expressing neurons, leads to a reduction in anxiety-like behavior [47]. This is especially relevant to the present study in which we demonstrate that ghrelin administration not only suppresses the activity of the pyramidal-like neurons in this area but also suppresses anxiety-like behavior in the EPM test when administered directly into the amygdala. Thus our electrophysiology data are supportive of the behavioral results, as ghrelin reduces both the activation of pyramidal-like neurons and anxiety-like behavior.

Previous studies determining the effects of intra-amygdala injection of ghrelin on feeding behavior are not altogether in agreement; whereas one study reported no effect on regular chow intake [19] another reported a decrease in liquid food intake [48]. In the present study we found that intra-amygdala injection of ghrelin robustly increased food intake in fed rats given free access to normal chow. Note that, in the present study, ghrelin was administered, by microinjection of a small volume, to the part of the amygdala where GHS-R is most abundant thereby limiting diffusion to adjacent structures (as depicted in Fig. 1). Given that lesions of the amygdala have been shown to increase or decrease food intake in a site-specific manner [49], we may infer that the focal site of ghrelin injection (including spread from the injection site) may be critical for determining the feeding response, and likely differs between these different studies.

Previously we demonstrated that central administration of a ghrelin receptor antagonist suppresses the hyperphagia observed after an overnight fast [33]. Given that ghrelin levels are high during fasting [50], providing a strong motivational drive for food intake [5]–[7], [39], these findings suggest that endogenous ghrelin could have a role in hunger-induced food intake. Here we extend these studies to demonstrate that food intake after an overnight fast can be reduced by administration of a ghrelin receptor antagonist directly into the amygdala. We may infer, therefore, that endogenous ghrelin signaling at the level of the amygdala likely contributes to feeding control, especially food intake and associated behaviors linked to caloric deficit.

The source of endogenous ghrelin in the amygdala remains an open question. The afferent ghrelin signal to the amygdala could be blood-borne [1], [51] but it could equally be derived centrally as a functional hypothalamo-amygdala ghrelin projection has been described, reflected by an increased number of amygdala cells activated (that express c-fos) after parenchymal administration of ghrelin into the lateral hypothalamus or the paraventricular nucleus [17], [52].

For survival, it seems rather logical that appetite-regulating peptides would also regulate neurobiological pathways involved in emotional reactivity/mood. Consistent with this, anorexigenic peptides such as leptin [53] and cholecystokinin [54] have been reported to increase anxiety-like behavior, whereas the orexigenic peptide neuropeptide Y [54] (an established target for ghrelin in the arcuate nucleus [55]) reduces anxiety-like behavior. Although an orexigenic peptide, several studies, conclude that ghrelin has anxiogenic properties, when administered either centrally [26] or into specific sites of the brain such as the hippocampus, amygdala, dorsal raphe nucleus [19] as well as in hypothalamic nuclei, with the arcuate nucleus and the paraventricular nucleus the more sensitive regions to evoke the anxiety-like response [29]. Indeed, in a previous study we also reported increased anxiety-like behavior in rats following chronic (>2 weeks) central (lateral ventricle) infusion of ghrelin, with parallel changes in the expression of a number of candidate genes in the amygdala [11]. In contrast to data showing anxiety-like effects of ghrelin, Lutter and colleagues reported that increasing ghrelin levels, either by caloric restriction or subcutaneous injections of the peptide in mice, elicits anxiolytic- and anti-depressant-like behavior in the elevated plus maze and the forced swimming test respectively. They also reported an increase in ghrelin levels in a rodent model of depression due to chronic stress. Accordingly, they suggest that an increase in ghrelin levels induced by a situation of stress would help to cope with the stressors [10].

In the present study, we sought to clarify the neurobiological effects of ghrelin on anxiety-like behavior focusing only on those exerted at the level of the amygdala, that we now know are relevant for feeding control and likely linked to emotional reactivity. We reasoned that the outcome of the anxiety testing could be dependent upon food availability when the ghrelin is administered (i.e. whether animals are able to eat or not after receiving the ghrelin injection). In other words, the afferent ghrelin hunger signal is expected to coordinate a feeding response but if no food is available, it could be advantageous for survival if emotional (anxiety-like) behaviors that would otherwise limit the animal from finding food are suppressed. To explore these emotional behaviors, we focused especially on anxiety-like behavior (in the EPM test and the open field) for which an advantageous decrease in anxiety-like behavior is signified by an increased time spent in the open arm or the centre field respectively. Thus, we performed the anxiety tests 1 hr after intra-amygdala saline/ghrelin injection in two different experimental paradigms, one in which the animals were allowed to feed between the injection and the anxiety test and another in which food access was denied. In the EPM test, ghrelin increased both the number of entries into the open arm and the amount of time spent there (relative to saline-injected controls), in rats that were not given access to food during the first hour after the injection. Conversely, in rats allowed to feed during the first hour after intra-amygdala injection, ghrelin had no effect on any parameter measured in the EPM test. These effects of intra-amygdala ghrelin on anxiety-like behavior were further validated in the open field test. Additionally, we were able to show that overall locomotor activity was similar for saline-injected and ghrelin-injected rats, both those with access to food and in those denied access to food during the hour after injection; thus any differences observed in these tests are not secondary to a locomotor effect of ghrelin in the amygdala. Thus, ghrelin's role at the level of the amygdala appears to include the suppression of anxiety-like behavior (including those that limit the rat from finding food) when food is not available. In the present study we did not determine whether food availability per se alters emotional (anxiety-like) behavior in the anxiety tests. Thus, in situations when food is not available, further studies would be required to determine whether ghrelin reduces anxiety-like behavior per se or whether it prevents an anxiogenic effect of withholding food.

Given the electrophysiological and neuroanatomical data identifying the amygdala as a target for ghrelin, it seems rather likely that the behavioral effects observed after intra-amygdala ghrelin administration involve a direct action at the level of the amygdala. We know very little about local concentrations of ghrelin after parenchymal injection to the amygdala, or about its half-life when delivered via this route. It is theoretically possible that ghrelin could even diffuse to other brain areas that could be important for the observed behavioral effects, such as hypothalamus or closer structures such as the hippocampus. In previous studies, however, involving parenchymal injection of ghrelin to a different brain area, the ventral tegmental area, no effects were observed when the peptide was delivered “off-target" to neighboring structures using doses similar to those in the present study [56].

In summary, our results provide neuroanatomical, electrophysiological and behavioral data indicating that the amygdala, especially the lateral nucleus (shown here to be the region with highest expression of the ghrelin receptor, GHS-R) is a key brain target for ghrelin, integrating effects of food intake and emotional reactivity. Our observation that ghrelin's acute effects to suppress anxiety-like behavior in the EPM test, exerted at the level of the amygdala, are only observed in rats that are prohibited from eating during the first hour after ghrelin administration, support the idea that ghrelin's effects on food intake and emotional reactivity are linked and likely facilitate exploration, foraging and other food-linked behaviors that are critical for survival when food is scarce.
